# A formative evaluation of the TB Think Tank’s role and influence on TB policy in South Africa

**DOI:** 10.1371/journal.pgph.0006229

**Published:** 2026-05-06

**Authors:** Bey-Marrie Schmidt, Chanelle Mulopo, Lilian Mayieka

**Affiliations:** 1 Health Systems Research Unit, South African Medical Research Council, Cape Town, South Africa; 2 School of Public Health, University of the Western Cape, Cape Town, South Africa; 3 Kenya Medical Research Institute, Nairobi, Kenya; London School of Hygiene & Tropical Medicine, UNITED KINGDOM OF GREAT BRITAIN AND NORTHERN IRELAND

## Abstract

Tuberculosis (TB) remains one of the leading causes of death globally, despite being both preventable and treatable. South Africa continues to experience a high burden, with an estimated 56,000 TB-related deaths recorded in 2023. In response to this public health crisis, the TB Think Tank (TB TT) was established in 2014 to strengthen the government’s programmatic response to TB. This formative evaluation examines the activities, processes, and outputs through which the TB TT has influenced TB policy between 2021 and 2024, identifies areas for improvement, and provides actionable recommendations to enhance its impact. To conceptualise the TB TT as a Knowledge Translation Platform, Bennett and Jessani’s (2011) framework was applied, focusing on knowledge generation, dialogue, and capacity building. Data were collected through 14 in-depth interviews with key stakeholders, a survey of 65 TB TT members conducted between June and July 2024, document review, and observational data to provide contextual insight; survey data were analysed using descriptive statistics, while qualitative data were analysed using inductive thematic analysis. Findings indicate that the TB TT has played a significant role in shaping South Africa’s TB response, with 97% of survey respondents affirming its effectiveness in advising the National Department of Health on policy. Key strengths include diverse membership, strong leadership, and alignment with national priorities, which have supported influential outputs such as the TB Strategic Plan 2023–2028. However, several challenges persist, including delays in the approval of recommendations, underrepresentation of key stakeholders such as district and provincial practitioners and other government sectors, limited monitoring mechanisms, and broader systemic constraints such as bureaucratic processes, competing priorities, and data access issues. Overall, the TB TT has made a substantial contribution to South Africa’s public health landscape and remains well positioned to advance the national TB agenda by strengthening evidence-informed decision-making and promoting equitable care.

## Background

Tuberculosis (TB) is an infectious disease primarily affecting the lungs and caused by the bacterium *Mycobacterium tuberculosis* [1]. Despite being preventable and treatable, TB remains one of the top ten causes of death globally, claiming approximately 1.5 million lives each year [1]. The disease disproportionately impacts low- and middle-income countries such as South Africa, where health systems often face significant challenges in controlling its spread [1]. Social determinants of health—including poverty, overcrowding, and malnutrition—further exacerbate TB transmission and adverse outcomes [ 2,3,Hargreaves et al., 2011[3] ]. TB control efforts are complicated by the emergence of multidrug-resistant (MDR-TB) and extensively drug-resistant TB (XDR-TB), which are more difficult and costly to treat. Additional obstacles include delays in early and accurate diagnosis, poor adherence to treatment regimens, and under-resourced health systems lacking adequate infrastructure, funding, and trained personnel to manage cases effectively [4,5].

Beyond these biomedical and health system challenges, a significant barrier to effective TB control is the limited use of research evidence in policy and practice decision-making [6]. Strengthening TB control thus requires deliberate knowledge translation (KT) mechanisms that ensure the timely, relevant, and context-specific synthesis, dissemination, exchange, and ethical application of research evidence to inform policy decisions and implementation processes [7]. However, effective KT is hindered by limited institutional support, insufficient financial and technical resources, gaps in skills and networks among researchers, negative attitudes or poor understanding of KT among evidence users, and a scarcity of locally relevant research to guide micro-level policy and practice decisions [8–11].

In response, there is growing recognition of the importance of sustainable knowledge translation platforms (KTPs) that bring together diverse health decision-makers—including policymakers, healthcare providers, patient representatives, and funders—institutionalise the use of research evidence in policy and practice decision-making [11,12]. KTPs act as intermediary organisations designed to overcome these challenges by promoting collaborative knowledge production, capacity building, information exchange, and ongoing dialogue [13,14]. By facilitating timely and meaningful engagement among diverse health decision-makers, KTPs play a vital role in bridging the persistent gap between research and policy.

The South Africa TB Think Tank (TB TT) was established in 2014 during the Fourth South African TB Conference as a strategic response to persistent gaps between TB research and policy [15]. It functions as a formal KTP, supporting the National Department of Health (NDoH) by synthesising, adapting, and facilitating the use of TB research in the policy decision-making processes of the National TB Programme (NTP). In addition, the TB TT operates as a network of TB experts committed to advancing innovative, evidence-informed solutions for TB prevention and control within the National TB Programme. It provides technical advice to the NTP on policy and programme development, fosters ongoing dialogue among stakeholders, and contributes to national strategic plans, guideline development, and monitoring frameworks [16].

The TB TT is structured around three main components: an Executive Committee (ExCo), a Secretariat, and a network of five task teams and two working groups. The ExCo provides strategic oversight, ensuring alignment with NDoH priorities, approving annual workplans, and coordinating with donors and implementing partners. Chaired by the NTP director, the ExCo includes members from the Secretariat and key partner organisations. The Secretariat, housed within the Aurum Institute, coordinates day-to-day operations, manages communication across task teams and working groups, and supports documentation and policy engagement. Since 2021, the Secretariat has included a full-time embedded scientist to strengthen evidence synthesis and facilitate engagement with decision-makers.

The work of the TB TT is operationalised through five task teams—Finding Missing People with TB, Optimising Treatment Outcomes, Data Systems and Innovation, TB Prevention, and TB Epidemiology, Modelling, and Health Economics—as well as two working groups: Child and Adolescent TB, and TB in the Mines. Task teams and working groups operate through consensus-based decision-making, producing outputs such as policy briefs, technical reports, and recommendations, which are submitted to the ExCo for endorsement and integration into NTP activities. This structure enables broad stakeholder engagement while maintaining strategic coherence with national priorities.

The TB TT is supported by a mix of domestic and external funding. The initial investment by the Bill and Melinda Gates Foundation (BMGF) was instrumental in launching the TB TT. This early support enabled the platform to gain momentum and attract further funding from the United States Agency for International Development (USAID) and the Centers for Disease Control and Prevention (CDC). Currently, the TB TT Secretariat is funded by USAID, CDC, the BMGF—which returned as a funder in August 2023 after a brief hiatus—and the South African Medical Research Council (SAMRC). These resources have supported key personnel, expanded coordination capacity, and ensured continuity across policy engagement cycles TB [15].

Today, the TB TT comprises approximately 380 members from a wide range of stakeholder groups. Over 280 members actively volunteer across its task teams and working groups, contributing to evidence generation and knowledge translation activities aligned with the NDoH’s annual priorities. Membership includes representatives from the NDoH, public-sector health professionals, researchers, implementing partners, donor agencies, civil society, and the private sector. This diversity enhances the platform’s ability to address TB and TB-HIV-related challenges comprehensively and collaboratively.

### Evaluating the TB Think Tank

White et al. [16] outlined key lessons learned during the establishment of the South Africa TB Think Tank (TB TT) between 2014 and 2016. They identified its core roles as an institution, a policy dialogue forum, and a research–policy interface supporting national TB decision-making. One early challenge was sustaining stakeholder engagement, which was most effective when activities aligned with the NTP’s priorities and timelines. The TB TT demonstrated tangible policy impact, contributing to South Africa’s first TB-HIV investment case and the creation of a ring-fenced TB grant. The paper emphasised the importance of structured coordination, focused workplans, and active participation from both programme staff and technical experts, concluding that the TB TT model could be adapted to other contexts to strengthen evidence-informed policymaking.

Nearly a decade later, the TB TT commissioned an external evaluation team (BMS, LM, and CM) to assess its characteristics, including its activities, processes, outputs, stakeholder engagement, strengths, limitations, and its overall role in translating TB evidence into national policy. This formative evaluation, conducted between 2021 and 2024, focused on the TB TT’s third iteration, launched in 2021. This phase of the TB TT marked a period of substantial reform, including a revised funding mechanism, the establishment of a new ExCo and Secretariat with the addition of a full-time embedded scientist, and changes in task team and working group leadership.

This formative evaluation examines the activities, processes, and outputs through which the TB TT has influenced TB policy between 2021 and 2024, identifies areas for improvement, and provides actionable recommendations to strengthen its role in combating TB in South Africa.

## Evaluation methods

To conceptualise and understand the TB Think Tank as a KTP, we drew on Bennett and Jessani’s [17] framework, which outlines three core components of KTPs: knowledge, dialogue, and capacity [17]. Knowledge refers to the role of a KTP in defining and identifying knowledge, and then harvesting, preparing, and synthesising it. Dialogue refers to the ability of a KTP to deliberately facilitate discussions around prioritized questions and topics. Capacity in the context of a KTP means providing evidence users with support in acquiring, assessing, adapting, and applying research in decision-making. This evaluation assessed the overall functioning and influence of the TB TT in translating TB evidence into national policy

### Data collection and analysis methods

This mixed-methods formative evaluation combined qualitative and quantitative approaches to explore participants’ views and experiences with the TB Task Team (TB TT). Primary data were collected through in-depth interviews and an online survey and supplemented by contextual information from internal documents and covert participant observations conducted during the TB TT annual meeting.

To provide verify and provide background to the primary data, two researchers (CM and LM) reviewed internal documents provided by the TB Think Tank Secretariat—including meeting notes and presentations—as well as publicly available materials on the TB TT website, such as webinars, newsletters, and technical reports. Covert non-participant observation was conducted by CM and BMS during the TB TT’s annual in-person meeting. They observed plenary sessions and task team discussions, focusing on interaction dynamics, while maintaining a non-intrusive presence and discreetly recording field notes.

Between 1 June and 31 July 2024, 14 in-depth interviews were conducted with TB TT stakeholders. Participants were selected from a list of executive committee, task team, and working group members using convenience sampling, with maximum variation to ensure diversity in gender, stakeholder group, length of involvement, and role within the TB TT (Table 1).

**Table 1:** Interview participants.

Participants	Gender	Task team/ working group	Duration
1	Female	TB Prevention	8 Years
2	Male	TB Epidemiology, Modelling and Health Economics	<10 Years
3	Female	TB Prevention	3 Months
4	Male	Data Systems and Innovations	2 Years
5	Female	Optimising Treatment Outcomes	7 Years
6	Male	Childhood and Adolescent TB	1-2 years
7	Female	Finding Missing People	2 Years
8	Female	Optimising Treatment Outcomes	<10 years
9	Male	Data Systems and Innovations	2 years
10	Female	TB Epidemiology, Modelling and Health Economics	8 years
11	Female	Data Systems and Innovations	7 years
12	Male	TB in the Mines	5 years
13	Male	Data Systems and Innovations	3 years
14	Male	TB in the Mines	<10 years

Invitations were sent by email, including study information, a consent form, and a request for availability. Interviews were conducted in English via Microsoft Teams, lasted 50–60 minutes, and were facilitated by two researchers—one leading the discussion and the other taking notes. A semi-structured guide with 14 open-ended questions (Box 1) was used. With participant consent, interviews were audio-recorded and transcribed verbatim.

**Box 1. Interview questions**

1. What is your role within the TB Think Tank?
2. What is the purpose, function, structure and model of the TB Think Tank?
3. How is the TB Think Tank funded? How are its operations sustained over time?
4. Who are the key collaborators and/or users of the TB Think Tank? What is their role?
5. Has the TB Think Tank changed over time (in activities, processes and outputs), e.g., from when it started to now, or before and after the COVID-19 pandemic?
6. What activities and processes are carried out within the TB Think Tank?
7. What kinds of outputs does the TB Think Tank produce?
8. How is evidence produced within the TB Think Tank used in policy and practice decision-making processes?
9. What is the TB Think Tank’s approach to strategically engaging relevant stakeholders, and disseminating and communicating evidence and outputs produced within the TB Think Tank?
10. What makes the TB Think Tank work well? What are its strengths? Can you share a situation or story.
11. What makes the TB Think Tank not work well? What are its weaknesses or limitations? Can you share a situation or story.
12. How can the work of the TB Think Tank be improved or strengthened?
13. Do you think that the TB Think Tank should be organised or positioned differently to advance its work?
14. Do you have processes in place to monitor and/or evaluate whether or how the TB Think Tank is successful in influencing policy and practice? Are you tracking outcomes at the individual and clinical level?

An online survey was developed in Google Forms and distributed by the TB TT programme manager to all members via email. The survey included 26 questions:14 closed-ended and 12 open-ended (see Box 2) on participant demographics and perceptions of TB TT activities and processes. The survey remained open for four weeks, with weekly reminders. A total of 65 members responded.

**Box 2. Survey questions**

1. What is your sex?
2. Which stakeholder group do you belong to?
3. Are you involved in any of the task teams and/or working groups?
4. Describe your role in the TB Think Tank.
5. How long have you been involved with the TB Think Tank?
6. On a scale of 1–5, how satisfied are you with your involvement in the TB Think Tank’s activities?
7. Do you feel that the TB Think Tank includes a diverse range of stakeholders in its discussions and decision-making processes?
8. If you answered ‘no’ to the question above, which stakeholder group(s) do you think is/are underrepresented?
9. On a scale of 1–5, how effective do you think the TB Think Tank has been in collating, reviewing, synthesising and evaluating evidence related to policy and implementation?
10. On a scale of 1–5, how effective do you think the TB Think Tank has been in soliciting or requesting evidence, and when needed research, to guide policy and implementation?
11. On a scale of 1–5, how effective do you think the TB Think Tank has been in identifying and prioritising research questions?
12. On a scale of 1–5, how effective do you think the TB Think Tank has been in advising the National Department of Health on policy implementation?
13. What does the Think Tank do particularly well?
14. What activities or initiatives of the TB Think Tank do you find most effective in producing evidence or influencing policy and practice? Please explain.
15. On a scale of 1–5, how satisfied are you with the quality of the evidence and recommendations produced by the TB Think Tank?
16. On a scale of 1–5, how satisfied are you with the quantity/frequency of the evidence and recommendations produced by the TB Think Tank?
17. Can you provide an example of a research output or recommendation that you found particularly influential, useful, or impactful in past year?
18. Do you think the TB Think Tank should be organised or positioned differently to advance its work?
19. If you answered ‘yes’ to the question above, how do you think it should be re-organised and re-positioned differently?
20. Do you think the TB Think Tank is prioritising the right things?
21. If you answered ‘no’ to the question above, how do you think the TB Think Tank can improve how it prioritises its work?
22. List internal and external barriers that affect the TB Think Tank’s functioning and impact.
23. In light of the barriers, you have listed above, how can the TB Think Tank be improved or strengthened?
24. List internal and external facilitators that affect the TB Think Tank’s functioning and impact.
25. Are there any new areas or issues you think the TB Think Tank should address in the future? Please explain.
26. Is there anything else you would like to share about your experience with the TB Think Tank? Please elaborate.

Survey data were exported to a secure, password-protected Excel file accessible only to the evaluation team. Closed-ended responses were summarised using descriptive statistics, and open-ended responses were analysed using narrative synthesis. Interview data were analysed thematically using [18]. An inductive coding process allowed themes to emerge directly from the data rather than applying a pre-existing framework such as Bennett and Jessani’s KTP framework. The evaluation team (BMS, LM, and CM) first reviewed transcripts for familiarisation. LM and CM independently coded the transcripts in ATLAS.ti (LM coded questions 1–7; CM coded 8–14), and BMS reviewed a sample of coded data for quality assurance. Codes were then grouped into themes based on shared patterns of meaning. Document and observation data were reviewed in parallel to provide contextual depth and support data triangulation.

### Ethics approval

Ethics approval for this study was obtained from the University of the Western Cape’s Biomedical Research Committee (reference number: BM21/10/26), as part of a larger study, *Knowledge Translation Platforms for Bridging Public Health and Health Systems Research into Universal Health Coverage-Related Policy and Practice in South Africa* (KTP-UHC), being conducted by the evaluation team.

## Evaluation results

### Stakeholder representation and involvement in the TB TT

The TB TT has a diverse membership including managers and programme implementers from various levels of the NDoH, academics and researchers, and representatives from funding agencies, non-government organizations (NGOs), civil society (e.g., South African National AIDS Council), and the private sector. Most respondents (77% of 65 respondents) said that the TB TT includes a diverse range of stakeholders in its decision-making processes. The remaining respondents (23% of the 65 respondents) said that some stakeholder groups were underrepresented, including district and provincial health practitioners (e.g., nurses and doctors), patient and advocacy groups, basic scientists, and representatives from other relevant government departments (e.g., information systems, education, social services) (see Table 2). Interview participants recognised the importance of having provincial implementers and patient and public advocates involved in the decision-making processes to ensure that policies are developed with practical implementation in mind.

**Table 2:** Close-ended survey questions and responses.

On a scale of 1–5, how satisfied are you with your involvement in the TB Think Tank’s activities?	Very satisfied 13/65 (20%) Satisfied 21/65 (32%) Neutral 18/65 (28%) Dissatisfied 7/65 (11%) Very dissatisfied 6/65 (9%)
Do you feel that the TB Think Tank includes a diverse range of stakeholders in its discussions and decision-making processes?	**Yes 50/65 (77%)** No 11/65 (17%) I am not sure 4/65 (6%)
On a scale of 1–5, how effective do you think the TB Think Tank has been in collating, reviewing, synthesising and evaluating evidence related to policy and implementation?	**Very satisfied 21/65 (32%)** Satisfied 19/65 (29%) Neutral 16/65 (25%) Dissatisfied 8/65 (12%) Very dissatisfied 1/65 (2%)
On a scale of 1–5, how effective do you think the TB Think Tank has been in soliciting or requesting evidence, and when needed research, to guide policy and implementation?	Very satisfied 20/65 (31%) **Satisfied 21/65 (32%)** Neutral 14/65 (22%) Dissatisfied 7/65 (11%) Very dissatisfied 3/65 (5%)
On a scale of 1–5, how effective do you think the TB Think Tank has been in identifying and prioritising research questions?	Very satisfied 14/65 (22%) **Satisfied 24/65 (37%)** Neutral 17/65 (26%) Dissatisfied 7/65 (11%) Very dissatisfied 3/65 (5%)
Do you think the TB Think Tank has been effective in advising the National Department of Health on policy implementation?	**Yes 63/65 (97%)** No 2/65 (3%) I am not sure 0/65 (0%)
On a scale of 1–5, how satisfied are you with the quality of the evidence and recommendations produced by the TB Think Tank?	Very satisfied 20/65 (31%) **Satisfied 26/65 (40%)** Neutral 10/65 (15%) Dissatisfied 7/65 (11%) Very dissatisfied 2/65 (3%)
On a scale of 1–5, how satisfied are you with the quantity/frequency of the evidence and recommendations produced by the TB Think Tank?	Very satisfied 12/65 (19%) **Satisfied 27/65 (42%)** Neutral 19/65 (29%) Dissatisfied 6/65 (9%) Very dissatisfied 1/65 (2%)
Do you think the TB Think Tank should be organised or positioned differently to advance its work?	Yes 12/65 (19%) No 26/65 (40%) **I am not sure 27/65 (42%)**
Do you think the TB Think Tank is prioritising the right things?	**Yes 53/65 (82%)** No 3/65 (5%) I am not sure 9/65 (14%)

Most survey respondents (83% of 65 respondents) were involved in a task team or working group and expressed that they were very satisfied (20% of 65 respondents) and satisfied (32% of 65 respondents) with their involvement in TB TT activities. Most members of the TB TT, besides the secretariat, fill multiple roles as contributors to and users of evidence and policy within and outside of the TB TT. For example, the chair of the TB TT is a contributor (he is involved in the *TB in the Mines* working group), while he is also a user (he uses outputs produced within the TB TT to inform his NDoH work).

Most participants reported that key outputs produced within the TB TT are for the NDoH and provincial implementers. One participant said: *“The biggest consumer of the Think Tank is the NDoH because it is the one who gives direction to the province, the clinics, and so on.”* Additionally, all members of the TBTT reported that they themselves, are users of the TBTT outputs.

### The role and function of the TB TT

Interview participants highlighted that the TB TT’s primary role is to generate and apply evidence for the development of national TB strategies. Most survey respondents (82% of 65 respondents) agreed that the TB TT is effectively prioritising the right topics and activities. This aligns with its complementary role in supporting the NDoH in combating TB, particularly through prioritising key areas and contributing to the development of strategic plans, such as the TB Strategic Plan 2023–2028. One interview participant described the TB TT as *“a platform for building consensus on how to improve policy and practice of the TB Programme nationally.”* A key function of the TB TT is to convene leading TB experts from various institutions to develop new policies, review existing guidelines, support policy-making, disseminate policies and guidelines, and address previously overlooked areas such as paediatric and occupational TB.

Another role of the TB TT is to generate evidence that informs policy. One participant explained: *“The main purpose is to be a knowledge and policy translation bridge or instrument... the success is that most of the policies proposed by the TB Think Tank are adopted by the National TB Programme.”* The TB TT serves as a platform for synthesising international and local research findings, assessing their applicability to the South African context, and providing the NDoH with evidence relevant to policy updates. Another participant emphasised that the TB TT “*puts these activities into place, generates the evidence which informs policy change,”* highlighting its role in translating research into actionable policies. The TB TT also packages evidence in a manner that facilitates easy understanding and adoption by the NTP. Participants generally supported this perspective, with one noting: “*If you just look at the amount and the speed at which guidelines are now released… these guidelines are not developed by one person; they undergo thorough reviews and rigorous critique.”*

Furthermore, the TB TT plays a critical role in setting the agenda for strategic plans and advocating for policy changes. By involving researchers, the platform aims to *“give researchers a more prominent role”* in shaping the priorities of the NTP, ultimately leading to more effective and responsive TB strategies. The TB TT also functions as a platform for sharing research findings and fostering collaboration among stakeholders. It excels in networking and stakeholder engagement, effectively bringing together diverse groups to collaborate and contribute to the fight against TB.

### Activities and outputs of the TB TT

Members of the TB TT actively contribute to a variety of activities within their task teams and working groups. These activities include attending meetings, synthesising existing evidence, conducting research, drafting and reviewing policies and guidelines, offering expert advice, presenting at conferences, and other related tasks. The TB TT produces various outputs such as webinars, academic peer-reviewed publications, newsletters, policy briefs, briefing notes, presentation slides, annual reports, standard operating procedures, and policy and guideline recommendations. The outputs are disseminated using various channels, including champions within the NDoH, stakeholder meetings, mailing lists, public webinars, institutional websites, and social media platforms, such as YouTube and LinkedIn.

Many survey respondents expressed high levels of satisfaction with the quality of evidence and recommendations provided by the TB TT, with 31% of 65 respondents reporting they were very satisfied and 40% reporting they were satisfied. Regarding the quantity of outputs, 42% of respondents indicated satisfaction, while 29% were neutral.

Survey respondents highlighted various examples of influential, useful, or impactful outputs over the past year, including the *Systematic screening for TB standard operating procedures*, the *Guidelines for the management of TB in children*, and the *TB Recovery Plan*. Interview participants echoed these sentiments, emphasising the significance of the TB TT’s activities and outputs. One participant noted: “*The Think Tank managed the drafting of the Vision 2028 policy, which is the national strategy for TB that has just been published… we also actually draft guidelines for the department, so the [Treatment of latent TB infection guidelines] were drafted in the Think Tank, and now the paediatric guidelines have been drafted in the Think Tank.”*

Furthermore, interview participants noted that the outputs of the TB TT were developed using evidence from diverse sources, including existing World Health Organization documents, available literature, targeted primary studies, and expert consultations. Researchers disseminated findings and facilitated new studies when additional evidence was needed: “*There were organisations that were actually coming to …being given tasks to do on behalf of the Think Tank, generate evidence, provide it, and this is how many of these guidelines were produced”*. This approach highlights the TB TT’s capacity to integrate diverse sources of evidence into coherent policy recommendations, ensuring that outputs are both scientifically robust and practically relevant.

### Evidence production for, and use in, TB policy-making

More than half of the survey respondents expressed high levels of satisfaction with the effectiveness of the TB TT. Specifically, 33% of the 65 respondents were very satisfied, and 29% were satisfied with how effectively the TB TT collates, reviews, synthesises, and evaluates evidence related to policy and implementation. Similarly, 31% were very satisfied, and 32% were satisfied with the TB TT’s ability to solicit and request evidence.. When it came to identifying and prioritising research questions, a third of respondents were satisfied (37%).

Notably, an overwhelming 97% of respondents agreed that the TB TT is effective in advising the NDoH on policy and implementation matters. Interview participants echoed these sentiments, emphasising the TB TT’s effectiveness in producing relevant and timely evidence to support the NDoH. However, all but one participant admitted they were unaware of how the evidence was used in decision-making after being shared with the NDoH. The only participant who could describe the process in detail explained that research used in TB policy must be protocol-driven, peer-reviewed, and published in an academic journal. The process begins by identifying relevant evidence syntheses from existing literature, and if none are available, new research is initiated.Experts are then consulted to provide insights, particularly when research is limited, conflicting, uncertain, or outdated. This iterative consultation process often involves multiple stakeholders, including NDoH officials, to approve the outputs.

Despite this systematic approach, one participant noted: *“…once our outputs are out and we have submitted them to the NTP, we don’t quite directly follow it up, but they are followed up by the cluster management.”* After expert consultations, research is consolidated into policy recommendations and submitted as tailored outputs to the NTP.

### Engagement, dissemination, and communication

Outputs are disseminated and communicated via the TB TT website, its mailing list, monthly newsletters, seminars, face-to-face meetings, webinars, social media, and TB conferences. The TB TT Secretariat requests researchers to report new scientific publications on a quarterly basis and provides opportunities for them to present their research findings to the broader platform. Outputs are also disseminated via civil society organisations across their networks. Additionally, the chair of the TB TT has a technical support unit at the NDoH that assists with disseminating outputs to provincial and district levels.

When it comes to stakeholder engagement, the TB TT is well-connected to the NTP via its chair, who is also the Chief Director of the NTP. Members of the TB TT participate in an annual face-to-face stakeholder meeting, as well as ad hoc task team and working group meetings. Most participants noted that district and sub-district practitioners and managers hardly attend these meetings unless they are directly involved in a project. One participant mentioned that: “*you’re not going to necessarily find a…some maybe, like a district or a sub-district TB manager necessarily, unless they’re involved in a project and they’re not the most frequent attendees”.* Nevertheless, other stakeholders, such as NDoH representatives and provincial managers are usually well-represented.

Interview participants highlighted the critical role of sustained donor funding in enabling the TB TT to support national TB policy development. Initial catalytic support from the BMGF helped establish the platform, while continued funding from USAID, CDC, and the SAMRC has ensured operational continuity and alignment with national priorities. However, several participants emphasised the need for the NDoH to eventually assume funding responsibility, given the TB TT’s close alignment with government objectives. As one participant explained: *“We had made an application to Global Fund to include it... but probably for sustainability, perhaps the government itself should consider starting to put some resources because it’s a structure that is very useful to the running of government services.”* Securing long-term sustainability through domestic funding was widely viewed as essential to maintaining the TB TT’s impact.

### Strengths of the TB TT

One of the TB TT’s key strengths is its strong buy-in and support from the NDoH and various reputable organisations. One participant affirmed this: “*I think it’s more of the enhanced support by the National Department of Health because I think that’s what gives the Think Tank its credence. You realise that the Think Tank doesn’t have more of a legal power on issues; rather, it has more of an influential power.”* Closely linked to this support is the strength of the TB TT’s leadership, particularly that of its chair, Dr. Ndjeka. Participants expressed high regard for his responsiveness and active engagement, which they credited with driving the TB TT’s increased output and effectiveness. One participant remarked: *“…I don’t think other countries have such a responsive TB director.”*

The TB TT also fosters a collaborative and informative environment that supports knowledge sharing, networking, and alignment around shared goals. As one participant described: *“It is a community that provides a space where one is kept up to date on key priorities in the TB community. It also provides opportunities to connect and network.”* Members are highly committed—even without financial incentives—and benefit from skilled leadership within task teams and working groups. This culture of collaboration contributes to the production of high-quality outputs, supported by a rigorous internal review process. As one participant highlighted: *“If you just look at the amount and the speed at which guidelines are now out… They are reviewed thoroughly, and there’s rigorous critique and review of those guidelines.”* This meticulous approach enhances the credibility, relevance, and impact of the TB TT’s work.

Another notable strength is the diverse representation of stakeholders within the TB TT. Members come from across the country, bringing varied experiences, perspectives, and professional backgrounds. This diversity enhances the representativeness and contextual relevance of the evidence and recommendations produced. Participants noted a marked improvement from earlier years—when membership was predominantly composed of white male clinical researchers—towards a more inclusive group in terms of ethnicity, gender, and culture. This shift has strengthened trust, transparency, and competence within the TB TT, further reinforcing its role as a valued partner to the NDoH.

Finally, participants emphasised the TB TT’s strong and respected reputation within the TB community. It is widely regarded as a credible and aspirational space for researchers and practitioners. This esteemed status is attributed to several factors: the TB TT’s ability to remain flexible and responsive to the local context, the effective leadership of its executive committee and secretariat, its capacity to attract and retain funding, and its long-standing presence over more than a decade. As one participant noted: *“The good part again is that we’ve been able to attract and retain some of these key stakeholders within the TB Think Tank. They are also involved in some of the activities, they are involved in some of the Task Teams, and there are people that also have vested interest in some of the work that we do.”*

### Limitations of the TB TT

While some task teams and working groups within the TB TT are functioning effectively, others face challenges in maintaining momentum and securing member commitment. A few participants noted these difficulties, with one stating: *“Last year, I just couldn’t get people to do anything, and I wasn’t the only task team that had that problem,”* and another commenting: *“People are just very busy.”* Although participation in the TB TT is voluntary, one participant raised concerns about the lack of compensation or recognition for members. The participant pointed out that while the TB TT receives significant funding from donors, much of which supports the secretariat, the sustainability of the TB TT’s work within task teams and working groups relies on unpaid contributions. Furthermore, the participant suggested that discontinuing certain task teams might improve resource efficiency.

In addition, some participants noted that the evidence produced by the TB TT is not always translated into suitable formats, and once submitted to the NDoH, it is not tracked for progress on implementation. One participant expressed frustration with this issue, saying: *“Having a guideline that doesn’t impact anyone on the ground—what is the value of a deliverable if it is not reaching the people on the ground? An algorithm exists but doesn’t impact every person walking into [Primary Care].”*

On the one hand, participants reported that producing evidence and developing guideline recommendations within the TB TT can be a slow process. One participant shared, *“On the prevention side, the [Treatment of latent TB infection] guideline took too long to complete, but it was done, and it was a useful guideline, though it didn’t move quickly enough.”* On the other hand, participants also noted that the uptake of evidence and the approval of guideline recommendations can be slow. For example, while the *Systematic screening for TB standard operating procedures* were developed within a year, its approval process was delayed. One participant expressed frustration with the approval procedures, stating: *“I find that process of approvals frustrating, but equally, sometimes I think that things sit at the Think Tank for too long, like the digital chest x-ray [standard operating procedure]… there could be a year where national policies are out, but provinces aren’t implementing them.”*

A few participants also pointed out that the low participation of provincial TB managers in TB TT meetings—where TB-related priorities are discussed, and decisions are made regarding policies and recommendations—negatively impacts the implementation of those policies at the provincial level. Another limitation of the TB TT is the challenge of balancing national TB priorities with the priorities of funders. One participant explained: *“At times it’s difficult to negotiate between what the real priority is versus what the funder is willing to fund; at times we find ourselves giving into the demands of the funder for something that maybe we would not have prioritized at that particular time.”* This highlights the complex dynamic the TB TT faces in managing expectations and potential conflicts of interest among stakeholders. As one participant noted, *“I just think we’re supposed to be independent, right? The funders are supposed to give us the funding to allow the task teams to decide what the priorities are. So, it feels like there are certain funders where that becomes quite challenging.”*

The bureaucratic nature of both the TB TT and NDoH was seen as a significant constraint, slowing down the efficiency of the TB TT’s activities. One participant noted, *“I think the main limitations are the bureaucracy—specifically in approvals—of some of the outputs. The process takes time, and it becomes unnecessarily long because it has to go through all those steps.”* Some participants expressed concern that the TB TT has not fully explored how to navigate the NDoH system effectively. They suggested that the secretariat explore the various forums within the NDoH where TB TT representation could contribute and establish appropriate feedback loops to keep members informed. However, they also acknowledged that this would place additional expectations on already-busy members, which could pose a challenge.

Another significant challenge faced by the TB TT is accessing relevant data from the NDoH. The NTP does not manage its own data, and although there have been ongoing efforts to engage with the division responsible for data management, these efforts have encountered numerous obstacles. One participant explained this challenge, saying: *“We are struggling with data management. We have a data management task team, but it’s struggling because there’s a much bigger problem with data management in the health services. We don’t have access to TIER data [the national electronic medical record system for drug-susceptible TB notifications]. If we want data from TIER, we need to request it from another department in the NDoH. We’re currently going through a whole review process to figure out how to interact with the reform process.”*

Furthermore, most interview participants reported a lack of understanding and clarity regarding the processes that follow the review of outputs within the TB TT and their subsequent submission to the NDoH. One participant said, *“That process is not very clearly defined, and if the process is clearly defined, it’s not very effective.”* Another participant noted the lack of communication about the approval process within the NDoH, stating: *“Because the guideline process is so drawn out, eventually you hear that something has been accepted, but it’s a bit convoluted. Nobody writes to the person who has presented it and says, ‘Thank you so much; it has gone to this stage,’ or even ‘Just to let you know, it has now progressed from stage one to stage two.’ There is none of that.”*

Participants also identified a dual challenge with the TB TT’s meeting structure. They felt that having only one in-person annual meeting was insufficient and suggested increasing the frequency to two meetings per year, as building relationships online proved difficult. However, they also found the frequent online meetings to be problematic, with some participants suggesting that certain communications could be managed via email. Coordinating these online meetings was another challenge, as finding suitable times that accommodate all interested individuals proved difficult. This issue underlined the need for a balance between in-person and virtual interactions to enhance both relationship-building and operational efficiency.

### Monitoring and evaluation of the TB TT

All participants reported that there are no formal, routine measures in place for monitoring and evaluating the influence and impact of the TB TT on national policy, aside from this and the previous evaluation. Additionally, there are no strategies for ongoing stakeholder engagement, dissemination, or community involvement. Task teams and working groups develop annual work plans, which the executive committee uses to check for progress of activities and outputs. The ExCo meets quarterly, during which task team and working group leads provide updates. One participant explained: *“We’ll have an ExCo meeting, and everybody will report, and it will be documented in the minutes, but that’s not what you’re talking about. You’re talking about a more formal monitoring and reporting process on the successes and failures.”*

However, implementing formal routine measures for monitoring and evaluating the TB TT could present challenges. One participant noted, “*I don’t want to force it too much into a rigid structure. We’re not an institution; we’re a forum of volunteers…a voluntary association of highly functioning people. The moment you start putting too many boxes and rules in place, it could work against you.”* This sentiment underscores the difficulty in balancing the need for accountability and evaluation with the flexibility and voluntary nature of the TB TT’s operations.

## Discussion, gaps, and lessons learned

This formative evaluation of the South Africa TB TT highlights the vital role of strategic initiatives, like KTPs, in narrowing the ongoing gap between research and policy in complex health systems. South Africa’s TB TT has emerged as a KTP bridging research and policy to advance TB control over the past decade. Its collaborative and inclusive approach has yielded influential outputs such as the TB Strategic Plan 2023–2028 and the TB Recovery Plan, underscoring its role in translating evidence into national policy. Comparable KTPs, such as EVIPNet Cameroon and REACH-PI Uganda, have similarly fostered evidence briefs, rapid syntheses, and policy dialogues, creating crucial spaces for expert deliberation on pressing health issues [19,20]. A key strength of the TB TT is its alignment with national TB priorities combined with diverse stakeholder representation, which enhances the relevance, credibility, and contextual fit of its outputs—consistent with global recommendations advocating broad engagement of evidence users to facilitate uptake [21].

However, this evaluation identifies persistent operational challenges common to many KTPs in LMICs [19,20]. Notably, there remains a lack of clear pathways to translate research into actionable policies and ensure implementation. Participants observed that policy briefs and recommendations frequently lack follow-up, weakening their influence. Bureaucratic delays within both the TB TT and NDoH further impede timely policy adoption, as illustrated by prolonged approval of key guidelines such as the Systematic Screening for TB SOP. Strengthening approval processes and enhancing feedback loops are critical to improving efficiency.

Stakeholder engagement also requires broadening, particularly to better include HIV specialists, public finance officials, and private sector actors. Their inclusion is vital to addressing the complex, intersecting determinants of TB and fostering multi-sectoral policy coherence. The TB TT’s reliance on voluntary participation raises concerns about sustainability and output quality, highlighting the need for formal recognition and resource support. Moreover, misalignment between the TB TT’s priorities and NDoH implementation needs, coupled with limited access to relevant data and suboptimal data management, undermines evidence generation and monitoring efforts. Enhanced collaboration between the TB TT and NDoH data divisions is essential.

A further gap is the absence of a comprehensive monitoring and evaluation framework to assess the TB TT’s impact on policy and practice. While quarterly task team monitoring exists, it falls short of capturing systemic outcomes or informing adaptive management. Embedding formal evaluation strategies with clear indicators and feedback mechanisms is recommended to bolster accountability and continuous improvement [22].

Finally, the TB TT’s reliance on donor funding—primarily from U.S.-based sources—renders it vulnerable to external funding fluctuations. The termination of U.S. funding in February 2025 has had immediate and substantial effects on the operational capacity of the TB TT. Until this point, the funding supported a significant portion of the TB TT’s secretariat staff, enabling full-time engagement in evidence-to-policy activities. Following the loss of this support, staffing capacity has been reduced, with further restructuring planned, including the merging of key roles and continued operation with reduced staff time. These changes threaten the TB TT’s ability to perform core functions such as coordinating the task teams and working groups, convening scientific webinars, producing rapid evidence syntheses, and supporting the implementation of national TB policies. This challenge is not unique to South Africa; similar initiatives in other low- and middle-income countries have also been affected by the termination of U.S. funding, while many others remain vulnerable due to their continued reliance on external donor support [23].

In summary, the TB TT has made substantial contributions to South Africa’s TB response by strengthening evidence-informed policy. Addressing identified operational and systemic challenges—especially those related to KT, stakeholder inclusion, monitoring and evaluation, and sustainable financing—will be essential to enhancing its effectiveness and long-term impact. Doing so can further establish the TB TT as a global exemplar for KTPs operating in high-burden, resource-constrained settings.

## Conclusion

This evaluation affirms the South Africa TB TT’s critical role in advancing evidence-informed TB policy through its collaborative, responsive, and nationally aligned approach. Its model exemplifies how structured engagement between researchers and decision-makers can drive impactful public health strategies in resource-constrained settings. Addressing persistent challenges—including limited research uptake pathways, stakeholder gaps, inadequate monitoring and evaluation, and financial sustainability—will be key to strengthening its long-term influence.

## Supporting information

S1 Checklist Checklist of inclusivity in global research. (DOCX)
