# Supplementary material for: A formative evaluation of the TB Think Tank’s role and influence on TB policy in South Africa
Source: PLOS Glob Public Health. 2026 May 6;6(5):e0006229. doi: 10.1371/journal.pgph.0006229 (PMC13148654; doi:10.1371/journal.pgph.0006229)
Supplement: S1 Checklist — (DOCX) [file pgph.0006229.s001.docx]

Inclusivity in global research

PLOS’ policy on inclusivity in global research aims to improve transparency in the reporting of research performed outside of researchers’ own country or community and ensures that PLOS publications reporting global research adhere to high standards for research ethics and authorship. Authors of relevant research articles may be asked to complete the questionnaire below, which outlines ethical, cultural, and scientific considerations specific to inclusivity in global research. This questionnaire may be requested when researchers have travelled to a different country to conduct research, if research uses samples collected in another country, research with Indigenous populations or their lands, or if research is on cultural artefacts. Researchers travelling to another country solely to use laboratory equipment will not normally be required to complete the questionnaire. However, the questionnaire can be requested at the journal’s discretion for any submission – if you have been requested to complete this questionnaire by the PLOS journal you submitted to, please do so.

Please complete the questionnaire below and include this as a Supporting Information file with your manuscript. Note that if your paper is accepted for publication, this checklist will be published with your article in the supporting information files. Please ensure that you reference the checklist in the main body of your manuscript. We suggest adding a subsection ‘Inclusivity in global research’ to your Methods section and adding the following sentence: “Additional information regarding the ethical, cultural, and scientific considerations specific to inclusivity in global research is included in the Supporting Information (SX Checklist)”

The questions have been designed to be applicable to a wide range of study types, and there are subsections for both human subjects research and non-human subjects research. If any of the questions are not relevant to your research please mark them as “N/A” as appropriate.

**Ethical considerations, permits and authorship**

*This section is applicable to all research types.*

Provide details as to who granted permissions and/or consent for the study to take place in the Methods section of your manuscript. This should include the names of **all** ethics boards, governmental organizations, community leaders or other bodies that provided approval for the study. If individuals provided approval refer to these people by their role or title but do not list their name(s).

Reported on page number:9

Ethics approval for this study was obtained from the University of the Western Cape’s Biomedical Research Committee (reference number: BM21/10/26), as part of a larger study on Knowledge Translation Platforms for bridging public health and health systems research into policy and practice in South Africa (KTP-UHC) (Page 9).

Participant anonymity and confidentiality were upheld throughout the study. Audio recordings and survey data were stored securely on password-protected devices accessible only to the evaluation team. No individual names were disclosed in any study outputs; instead, participants were identified by role or stakeholder group to protect their privacy. Audio recordings of the interviews were stored on a password-protected computer, while survey results were extracted from Google Forms into a Microsoft Excel spreadsheet, both accessible only to the evaluation team (Page 10).

If there were any deviations from the study protocol after approval was obtained please provide details of these changes in the Methods section of your manuscript.
Did this study involve local collaborators that are residents of the country where the research was conducted or members of the community studied? If you do not have any authors from said communities, please provide an explanation for this below. Everyone listed as an author should meet PLOS’ criteria for authorship and all individuals who meet these criteria should be included in the author byline, rather than the acknowledgements. For further information please see the journal’s Authorship Policy.

Reported on page number:

There were no deviations from the protocol.

**Human subjects research (e.g. health research, medical research, cross-cultural psychology)**

This study involved collaborators based in South Africa, specifically the Secretariat of the TB Think Tank. Although the TB Think Tank commissioned the study, its members were not included as co-authors to minimise potential bias in the evaluation. All listed authors meet PLOS authorship criteria, and no eligible contributors were excluded.

Did you obtain written informed consent from a representative of the local community or region before the research took place? How did you establish who speaks for the community? Details of written informed consent obtained from study participants should be reported separately in the Methods section of your manuscript.

The study was approved by the Executive Committee of the TB Think Tank. Participation in interviews and surveys was voluntary, with all participants providing informed consent prior to data collection. The consent process included sharing a study information sheet which explained the study objectives, procedures, risks, and benefits. Participants were asked to provide three potential dates for interviews, which were then scheduled approximately two weeks after the invitation (Page 6).

How did members of the local community provide input on the aims of the research investigation, its methodology, and its anticipated outcome(s)?

The TB Think Tank commissioned this study to evaluate its activities, processes, and outputs. During the bidding process, we drafted a protocol outlining the proposed aims, methodology, and anticipated outcomes, which was subsequently reviewed and refined by the TB Think Tank’s Executive Committee.

When engaging with the local community, how did you ensure that the informed consent documents and other materials could be understood by local stakeholders?

Members of the TB Think Tank are science-literate, and English is the primary language of communication. The consent process included providing a study information sheet and explaining the study’s objectives, procedures, risks, and benefits. An email was sent with the information sheet and consent form, and participants were asked to provide three available dates for interviews, which were then scheduled approximately two weeks after the invitation (Page 6).

Will the findings of the research be made available in an understandable format to stakeholders in the community where the study was conducted (e.g. via a presentation, summary report, copies of publications, etc.)? Please provide details of how this will be achieved.

Results were shared with members of the TB Think Tank through a tailored summary report and a webinar presentation. The final publication will also be made available in open-access format to ensure broader accessibility.

**Non-human subjects research using specimens/ animals collected as part of the study, or those housed in archival collections. Examples include archaeology, paleontology, botany and zoology.**

Did the permission you obtained from a local authority to perform the study include an agreement on access to outputs and benefit sharing? This may include procedures to enable fair distribution of the benefits and resources arising from the research performed. Please include any details of Prior Informed Consent and Benefit Sharing Agreements obtained. These may be required by field-specific regulations, for example the Convention on Biological Diversity (CBD) and the associated Nagoya Protocol.

The permission obtained to conduct this study did not require a formal Prior Informed Consent (PIC) or Benefit-Sharing Agreement, as the research did not involve biological specimens, genetic resources, or materials covered by the Convention on Biological Diversity (CBD) or the Nagoya Protocol. Instead, the study evaluated the functioning of the South African National TB Think Tank through the involvement of human participants. Benefit sharing was ensured through non-material means, including the dissemination of findings to the TB Think Tank secretariat and stakeholders, as well as the open-access publication of results to ensure broad accessibility.

If the material used in your study was imported, please A) provide the year it was imported and B) indicate whether permits were obtained to import/export the materials used, C) provide details of any permits obtained. If this information is not available, please indicate this.

No materials were imported for this study; therefore, no import or export permits were required or obtained.

If you used archival specimens, please state how the material used in your study was acquired by the institute it is held in and provide details of any permits obtained for the original excavations/ sample collection. If this information is not available, please indicate this.

This study did not involve archival specimens, archaeological samples, or other physical collections; therefore, no permits for excavation or sample collection were required.

How was the potential cultural significance of the materials collected in your study to local communities considered in your research design? Were Indigenous peoples and/or local researchers and institutions involved with archaeological excavations / collection of specimens? If so, please provide a description of their involvement.

The study evaluated the functioning and impact of the South African National TB Think Tank as a knowledge translation platform and did not involve archaeological excavations, the collection of physical specimens, or culturally sensitive materials from local communities. Consequently, issues of cultural significance to Indigenous peoples or local communities were not applicable to the research design.

If your manuscript includes photographs of human remains please indicate whether authors obtained permission from descendants or affiliated cultural communities to do so.

The manuscript does not include any photographs of human remains.
